# Market analyses of livestock trade networks to inform the prevention of joint economic and epidemiological risks

**DOI:** 10.1098/rsif.2015.1099

**Published:** 2016-03

**Authors:** Mathieu Moslonka-Lefebvre, Christopher A. Gilligan, Hervé Monod, Catherine Belloc, Pauline Ezanno, João A. N. Filipe, Elisabeta Vergu

**Affiliations:** 1 MaIAGE, INRA, Université Paris-Saclay, Jouy-en-Josas 78350, France; 2 Department of Plant Sciences, University of Cambridge, Downing Street, Cambridge CB2 3EA, UK; 3 AgroParisTech, Paris 75005, France; 4 INRA, UMR1300 Biologie, Epidémiologie et Analyse de Risques en santé animale, CS 40706, Nantes 44307, France; 5 LUNAM Université, Oniris, Ecole nationale vétérinaire, agroalimentaire et de l'alimentation Nantes-Atlantique, UMR BioEpAR, Nantes 44307, France; 6 Integrative Animal Science, School of Agriculture, Food and Rural Development, Newcastle University, Newcastle upon Tyne NE1 7RU, UK

**Keywords:** animal trade networks, disease prevention, economic epidemiology, livestock, multiple-criteria decision analyses, risk-based surveillance

## Abstract

Conventional epidemiological studies of infections spreading through trade networks, e.g. via livestock movements, generally show that central large-size holdings (hubs) should be preferentially surveyed and controlled in order to reduce epidemic spread. However, epidemiological strategies alone may not be economically optimal when costs of control are factored in together with risks of market disruption from targeting core holdings in a supply chain. Using extensive data on animal movements in supply chains for cattle and swine in France, we introduce a method to identify effective strategies for preventing outbreaks with limited budgets while minimizing the risk of market disruptions. Our method involves the categorization of holdings based on position along the supply chain and degree of market share. Our analyses suggest that trade has a higher risk of propagating epidemics through cattle networks, which are dominated by exchanges involving wholesalers, than for swine. We assess the effectiveness of contrasting interventions from the perspectives of regulators and the market, using percolation analysis. We show that preferentially targeting minor, non-central agents can outperform targeting of hubs when the costs to stakeholders and the risks of market disturbance are considered. Our study highlights the importance of assessing joint economic–epidemiological risks in networks underlying pathogen propagation and trade.

## 1. Introduction

Trade is crucial for the economy, but can also drive infectious disease transmission, sustaining epidemics locally and promoting potentially long-distance introductions (e.g. [1]). Examples of markets that can contribute to epidemic outbreaks include trade of livestock such as cattle [2], swine [3], and sheep [4]; prostitution [5]; and airline transportation [6]. In the epidemiological literature, the contact structure underpinning pathogen spread through trading contacts is usually described using network models (e.g. [7]). In such models, holdings (e.g. farms) are represented by nodes that are interconnected by links that represent exchanges among holdings (e.g. movement of animals). In the past decade, network-based models have become increasingly popular as means to achieve a threefold objective: (i) to describe the contact structure spanned by such markets (e.g. [1]), (ii) to assess the epidemic risk factors at the scale of individual holdings (e.g. [8]) and (iii) to design effective disease control strategies (e.g. [9]). In particular, network analyses have proved useful in identifying super-spreading holdings, usually referred to as ‘hubs’, that should be preferentially subjected to trade restrictions in order to prevent and mitigate epidemics. The disruption of such core market players, however, can cause economic shocks, a downside, which, to the best of our knowledge, has not been considered in network-based, data-driven epidemiological studies. Here, we consider the key, but yet unaddressed question of evaluating the trade-off between the commercial efficiencies of trade routes and their vulnerabilities as routes for the transmission of economically damaging pathogens. We investigate this trade-off for livestock-exchange markets in France, for which we have access to extensive data.

The construction of network models of livestock markets requires the use of records of animal movements. In the European Union (EU), for instance, exhaustive tracing of livestock movement is available in many nationally maintained datasets, originally for cattle since the 1990s (Council Directive 92/102/EEC of 27 November 1992 on the identification and registration of animals) and more recently for swine (Council Directive 2008/71/EC of 15 July 2008 on the identification and registration of pigs). In other countries, data with such levels of detail may not be available because, for example, the data may have been aggregated to comply with privacy laws, or routine data collection may not be implemented at the farm level. The architecture of animal movements is often extremely rich and can be described by more or less simple network models depending on the objective of the epidemiological study [7]. In some cases, livestock-exchange network models can account for: more than one type of node (e.g. farms versus purely commercial holdings; e.g. [10]), direction of exchange (animals are essentially shipped from selling to buying holdings; e.g. [11]), weight of shipments (when the number of animals shipped in one go varies; e.g. [1]), and dynamical aspects (the shipment of animals occurs at certain points in time; e.g. [7]). It follows that empirical livestock-exchange networks exhibit key features shared by many complex networks, namely they are *multipartite*, *directed*, *weighted* and *dynamic*. Recent studies with network analyses of livestock markets provide significant information on demographic aspects and vulnerability to pathogen transmission in cattle and pig markets in several countries, especially where detailed data are routinely collected, including cattle, for example in the UK, Sweden, France and Italy [1,3,8,10] for cattle and Sweden, France and Germany [3,12,13], for pigs. There is increasing concern for the vulnerability of livestock systems with very large-scale connectedness, for example, as a result of open livestock trade among national markets such as the trade among the 27 member states of the EU [14], which includes movement of cattle, pigs, sheep, goats, poultry and horses. There have also been attempts at assessing the accuracy of epidemiological predictions in cases where detailed records of livestock movement are not available [15].

Various determinants of the risk of transmission of infection, and hence disease, can be calculated depending on the features included in a given network model. These determinants of risk range from the total number of commercial partners per agent (the total degree, e.g. [10]), the number of premises that can be reached through successive temporally compatible links [3] and the ability of each holding to preserve its commercial partners over time (an ability referred to as ‘loyalty’ [16]). In the general case, any measure of network centrality for a given holding can be used as an indicator of the corresponding risk of contagion. By identifying potentially highly contagious nodes, network analyses can inform the effective prevention and control of infectious disease transmission. It is general wisdom that such infectious ‘hubs’ should be targeted preferentially by the regulator (the public authorities enforcing health policy, which sometimes precludes such hubs from exchanging) in order to prevent and mitigate infectious disease outbreaks in exchange network systems [17–20].

While the implementation of trade restrictions on key large-size holdings could be effective in mitigating epidemic outbreaks, it is often prohibitively costly to regulators and has potentially severe economic impact on markets. Specifically, the disruption of core market players through intensive preventive measures can cause economic shock and render such strategies inappropriate [21]. The promising alternative of combining evaluation of economic and epidemiological risks, however, remains a key gap in the literature [22]; this is so, despite recent efforts mostly confined to theoretical studies based on models coupling epidemiological dynamics and economic aspects related to trade [23]. This gap is also related to the absence of well-established measures of cost effectiveness for managing livestock diseases [24].

Because the agents that are central to the market are also likely to act as sources for epidemic spread, we expect that in general there will be a strong association between economic and epidemiological risks, some of which may be negative (inverse) associations. In this study, we aim to identify efficient strategies for preventing epidemics with minimal disruption to markets and limited cost to the stakeholders such as regulators and business owners. We introduce a market-based categorization for aggregating the holdings based on economic as well as structural network summaries, namely *position along the supply chain* and *market share or leadership*. To study the economic–epidemiological implications of our categorization, we analyse two datasets recording cattle and swine livestock movement in France during 2005–2009 and 2010, respectively (§2). We show that the market categories that we propose describe livestock exchange intuitively and provide insights on the underlying trading patterns (§3.1). Using these categories, which are easy to implement because they are empirically defined, we evaluate the joint economic–epidemiological risks of epidemic outbreak and associated regulatory measures (§3.2). We consider both the regulator's and the market's standpoints and evaluate the effectiveness of different preventive strategies that target agents in selected market categories (§3.3). Both static and dynamical preventive strategies are explored, whether based on real-time or past data. We conclude by summarizing our most important results and by highlighting some perspectives for future work (§4).

The principal contribution of our study stems from adopting data-driven epidemiological and economic standpoints in order to evaluate control strategies against pathogen spread in livestock trade markets. Our study also contributes to the literature on network epidemiology by identifying how the choice of optimal outbreak control strategies may depend on the system considered (here cattle and swine livestock exchanges). Specifically, the optimal strategy identified does not necessarily rely on preferentially targeting hubs, despite the latter being often regarded as an evidently best approach.

## 2. Material and methods

### 2.1. Trade networks and livestock exchange

Describing livestock exchange from a market-centric perspective requires a preliminary exposition of core concepts, at the crossroad of economics and network theory. After a brief introduction to markets, we present the livestock-exchange data that we analyse as trade networks.

#### 2.1.1. Understanding markets from a network-centric perspective

As a first approximation, a market can be formally described as a network composed of economic agents (e.g. individuals, businesses or sovereign states) in interaction [25,26]. From a network perspective, an agent corresponds to a node or vertex interacting with other nodes through links or edges. From an economic perspective, an agent is an entity that pursues its own interests through some kind of economic optimization. Agents have generally divergent interests resolved through exchanges and price definition [27].

Based on [28], we define some core concepts to describe markets and their influence on epidemics. A market is made of supplying agents, i.e. *suppliers*, and demanding agents, i.e. *demanders*. Agents that are both supplying and demanding correspond to *wholesalers*. Agents interact during *transactions* by exchanging goods that can lead to disease transmission, where a transaction is a delivery from a supplier to a demander. *Trade flow* is the number of products (e.g. animals) traded from a supplier to a demander per unit time. Provided that trade is the only route for transmission, trade flow can be interpreted as the epidemiological contact rate (number of transactions per time unit) weighted by contact intensity (number of products exchanged per transaction).

Since the exchange of animals occurs from suppliers to demanders but often not in the reverse direction, and since different numbers of animals are shipped per transaction, we say that exchanges are *directed* and *weighted*. It follows that trade flow has a direction, e.g. we can dissociate *in-* and *out-*trade flows. We can also calculate the *total-*trade flow, i.e. the sum of the in- and out-trade flows. Hence, markets are described by directed and weighted networks. Moreover, since exchanges occur at precise points in time, markets form *dynamical* networks. Although more realistic, dynamical networks are harder to analyse than static networks (obtained, for instance, by aggregating interactions over time). In particular, the probability of outbreak emergence and the resulting impact on a trade network are more difficult to assess in time-varying than in static networks [29]. Here, we consider both types of networks.

#### 2.1.2. French livestock-exchange data described as trade networks

We analyse and compare trade networks derived from two datasets recording livestock exchange in France: the BDNI for cattle (managed by the French Ministry in charge of Agriculture (FMA)) over years 2005–2010, and BDPorc for swine (managed by the French professional union BDPorc) in 2010. Each dataset details movements of animals occurring in France among all economic agents involved in the supply chain, from strictly breeding farms to slaughterhouses with various categories of structural wholesalers in between (e.g. breeding–fattening farms, strictly fattening farms, dealers). Data on imports and exports are also available. Traceability is imposed by the regulator at different scales: on individual animals in the case of cattle, and on batches (sets of animals shipped from a seller to a demander during a transaction) in the case of swine. Hence, we extract individual-level transactions directly from the cattle dataset. Moreover, we reconstruct individual-level transactions for pigs based on a simple matching process (see the electronic supplementary material, section B.2 of [28], for details).

Our core network-based analyses are carried out at the microeconomic business scale, i.e. agricultural holdings, as far as national livestock exchange is concerned. The datasets allow us to distinguish three groups of holdings: *farms*, i.e. agents aiming to produce livestock; *trading agents* such as assembling centres, i.e. agents aiming to exchange livestock; and the *rest of the world*, a single entity aggregating all agents located outside of France, which we use to assess the importance of international restrictions on trade, as e.g. might occur in the case of a major outbreak. Following the epidemiological literature [3], we neglect slaughterhouses and movements to slaughterhouses from our analyses since including these movements would underestimate the risk of transmission associated with farms. However, we do include foreign movements to and from France as they can contribute to disease introduction, further dispersion on large geographical scales and major economic disruptions. Though we explored several temporal descriptions of networks (see the electronic supplementary material), all analyses presented in the main text are carried out on static networks aggregating transactions at the yearly scale for the sake of simplicity.

### 2.2. Market-centric categorization of economic agents for representing the trade networks

We introduce a generic categorization of economic agents applicable to a variety of markets including livestock exchange. We sort agents according to two types of market summaries: *position along the supply chain* and *market leadership*, and then use these categories to define *market categories*. Let 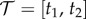 represent the period of time over which we aggregate the transactions. We then use these aggregates to calculate the following summaries.

#### 2.2.1. Position along the supply chain: flow polarity

Position along the supply chain is given by the overall direction of trade flow, that we quantify by a summary referred to as *flow polarity* and denoted fp*_a_*, and that is given for any agent *a* by the difference of its in- and out-trade flows divided by its total-trade flow over a particular time period 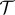:2.1
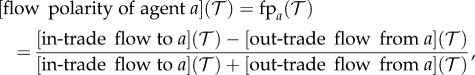
where [in-trade flow to *a*] (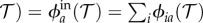 and [out-trade flow from *a*] (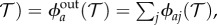 with 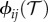 the trade flow from *i* to *j* over 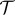 and where sums are over all nodes exchanging with *a* over the same period. By construction, 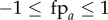 (2.1) and fp*_a_* can take any value between these two extremes. In order to build discrete classes of agents based on flow polarity, we introduce an empirical threshold *ε* > 0 which can take either predetermined values or be set equal to percentiles of a given distribution. Hence, agents *a* for which 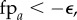 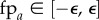 and 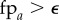 correspond to *suppliers*, *wholesalers* and *demanders*, respectively. Flow polarity is an extension of the concept of *hierarchy* [30,31] to weighted and dynamical networks.

#### 2.2.2. Market leadership: flow share

Following marketing studies [32], we use flow share, i.e. the relative trade flow, to quantify *market leadership*. For any agent *a*, flow share, denoted fs*_a_*, is defined as its total-trade flow over time period 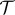 divided by the sum of total-trade flow for all agents over 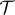:2.2
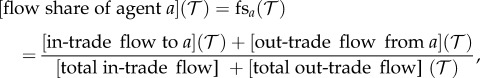
where 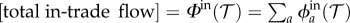 and 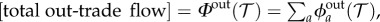 with sums over all active agents over 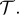 Flow conservation implies that 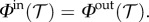 By definition, 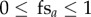 (2.2) and 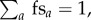 where the sum is over all active agents in the market during 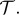 Similarly to flow polarity, we introduce two empirical thresholds 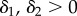 which can take either predetermined values or be set equal to percentiles of a given distribution. Agents *a* for which 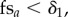 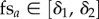 and 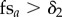 are denoted *nichers*, *followers* and *leaders*, respectively.

#### 2.2.3. Definition of market categories using flow polarity and flow share summaries

*Market categories* are defined based on a two-dimensional indicator (fp*_a_*, fs*_a_*), i.e. by the combination of position along the supply chain and market leadership: suppliers-nichers (SN), suppliers-followers (SF), suppliers-leaders (SD), wholesalers-nichers (WN), wholesalers-followers (WF), wholesalers-leaders (WL), demanders-nichers (DN), demanders-followers (DF) and demanders-leaders (DL). In addition to the categorization in 3 × 3 classes (with respect to the above definitions for fp*_a_* and fs*_a_*), finer grids can be adopted for more detailed analysis.

### 2.3. Elaboration, choice and evaluation of targeted control strategies

Based on the categorization of agents that we have introduced, we consider preventive strategies that involve preferential targeting of agents belonging to certain market categories. We evaluate generic forms of interventions for outbreak control. Specific practical examples of these interventions include preferential surveillance and vaccination of the agents that are deemed most at risk. We proceed in three steps: firstly, we elaborate a general class of strategies preferentially targeting agents belonging to specific market categories; secondly, we identify meaningful targeting strategies by assessing which agents are most at risk according to network-based summaries of economic and epidemiological risks; thirdly, we evaluate indirectly, from an economic–epidemiological perspective, how selected strategies influence systemic risk.

#### 2.3.1. Preferential targeting of agents in specific market categories

Let *N* denote the number of agents involved in at least one trade event during the time interval 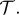 The fraction *F_n_* = *n*/*N* of *n* agents to be targeted is chosen according to strategy 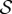 (scenario denoted 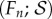), which ranks each agent *a* from 1 to *N* according to the decreasing values of a rank function, denoted 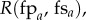 and based on the market categories as defined by fp and fs. We define 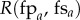 as the product of functions 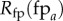 and 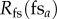:2.3
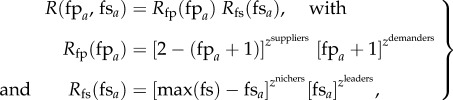
where *z*^suppliers^, *z*^demanders^, *z*^nichers^ and *z*^leaders^ ≥ 0 are fixed parameters representing the preferences of the regulator for targeting specific market categories. As an example, wholesaling leaders are surveyed preferentially when we set: *z*^suppliers^ = *z*^demanders^ > 0, *z*^nichers^ = 0 and *z*^leaders^ > 0. In the case when two or more agents take the same value of *R*, we choose their relative orders uniformly at random. Note that the variability resulting from this ranked ordering is weak, as confirmed by assessments of each strategy over 100 random replicate targeting for both datasets.

#### 2.3.2. Identification of specific targeted control strategies

To choose meaningful preventive strategies, i.e. to set *a priori* appropriate values for *z*^suppliers^, *z*^demanders^, *z*^nichers^ and *z*^leaders^ in (2.3), we calculate various risk indicators per agent per market category. We specify for each indicator whether it quantifies an *economic risk*, an *epidemiological risk* or *economic–epidemiological* *risks*. An *economic risk* is the risk of market disruptions caused by the failure of an agent. An *epidemiological risk* is the risk for a healthy agent to become contaminated and/or the risk of an infected agent to further transmit an infection to other agents. *Economic-epidemiological risks* are combined risks. In identifying optimal interventions, we are specially interested in strategies that minimize both economic and epidemiological risks.

First, we consider three risk indicators at the agent level: flow polarity, flow share and trade flow (defined in §2.1 and 2.2). We consider flow polarity is a measure of joint economic–epidemiological risks, flow share constitutes a measure of economic risk and trade flow quantifies epidemiological risk (although the relationships between these measures are subject to economic and epidemiological reinterpretations) [28,33,34]. We also calculate, for each market category, the values of two additional indicators of epidemiological risk that are well documented in the network epidemiology literature: the *proportion of agents belonging to the largest strongly connected component* (LSCC) and the *average betweenness centrality.* The LSCC is the largest subnetwork of agents for which a directed path exists from any other agent in the subnetwork. The betweeness centrality of a node is the fraction of shortest paths that passes through this node (see electronic supplementary material, section A.1 for details).

#### 2.3.3. Evaluation of the targeted control strategies using multi-criteria decision analyses

To evaluate targeting strategies, we carry out multi-criteria decision analyses (MCDA) from an economic–epidemiological perspective. The MCDA aim to find optimal strategies for reaching one or multiple objectives with minimal efforts, potentially considering multiple objectives and types of effort simultaneously [35]. The capacity of a strategy for mitigating a disease is measured through *prevention-effectiveness* criteria, while the effort needed for reaching a given effectiveness is measured through *prevention cost* criteria, where all criteria are to be defined. An optimal strategy is one that maximizes prevention effectiveness with minimal efforts. The strategies considered in this study are implemented by the regulator who decides to target certain agents preferentially. In practice, the cost of implementing control measures may fall on the regulator, the business owners or other stakeholders of the market. However, the perception of what is an optimal strategy is subjective. We hence consider two complementary points of view when comparing control strategies: the regulator's and the market's.

##### 2.3.3.1. Prevention effectiveness: derivation from the LSCC

We define prevention effectiveness as the benefit to the market stakeholders of implementing a preventive strategy, for instance, the losses averted by avoiding an epidemic. The proportion of agents belonging to the LSCC is a standard epidemiological proxy to assess both the probability of disease invasion and the epidemic final size [12,36]. Following node percolation experiments [17], we measure the prevention effectiveness of a given strategy 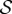 when targeting a proportion *F_n_* of agents by the *relative decrease in the LSCC size* of the network aggregated over the time period 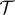:2.4
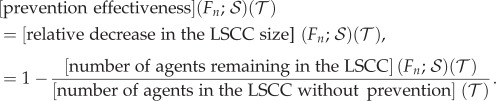
To assess the robustness of our findings based on the LSCC, we also use, in the electronic supplementary material, the average infection chain as an alternative proxy for prevention effectiveness [2,3,11]. In contrast with the LSCC, the average infection chain has the advantage of taking into account the sequence of dates when exchanges occur over time period 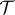 (and hence of considering only temporally compatible paths between agents). However, because of the considerable computation time required, calculation of the average infection chain is only performed for rather small networks. We also explore the influence of postponing the implementation of a given strategy 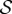 (see the electronic supplementary material, section A.2 for more details).

##### 2.3.3.2. Prevention costs: relative costs to the regulator and market distortions

We define multiple prevention costs, as the *relative costs to the regulator* and consequent *market distortions*. *Relative costs to the regulator* are costs incurred by the regulator when, e.g. implementing a preventive control strategy. *Market distortions* are potential damaging impacts on the market that result directly or indirectly from implementation of a preventive control strategy.

First, we detail relative costs to the regulator. When implementing a strategy 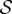 over time period 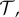 we assume the regulator may incur two types of costs: an *agent cost* and a *flow cost*. In common with others [17], though explicitly rather than implicitly, we assume the agent cost is proportional to *F_n_* for any strategy 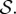 The flow cost is also expected to increase with *F_n_* but to be highly variable depending on the underlying strategy 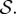 Here, in line with [28], we assume the flow cost is proportional to 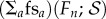 (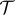), which is the total flow share of the fraction *F_n_* of agents targeted in strategy 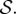 In practice, since we are interested in ranking a set of strategies, we measure *relative rather than absolute costs*: we hence directly track *F_n_* and 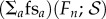
(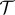), which take values in [0,1], as proxies for economic risk.

Second, we specify market distortions. Any preventive strategy implemented by the regulator is expected to cause market disruptions. Here, by analogy with our market categories, we consider two types of economic risk proxies to measure disruptions to the market: *disruption to the overall flow polarity* and *disruption to the overall flow share*. *Disruption to the overall flow polarity*, denoted 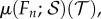 is measured as the relative mismatch between overall in- and out-flows:2.5
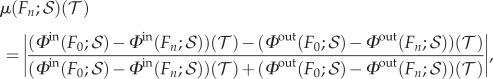
where 
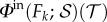
 and 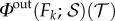 are the total in- and out-flows distorted when a fraction *F_k_* of agents are targeted according to strategy 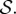 As specified in §2.2.2, flow conservation implies 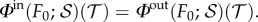 By construction, 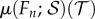 takes values in [0,1]. *Disruption to the overall flow share* is assessed by 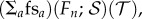 the total flow share targeted and hence potentially disrupted when a fraction *F_n_* of agents is targeted according to strategy 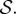 Note that the same value of 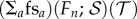 is used as a proxy for two differing quantities: flow cost (incurred by the regulator while implementing a strategy) and disruption to the overall flow share (which reflects a particular market disruption induced by the regulator's intervention).

##### 2.3.3.3. Practical implementation of the MCDA

Multiple criteria decision analyses are carried out over time period 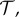 which we set to start in 2009 for cattle and 2010 for swine. To account for potential delays in the collection of data necessary to calculate flow polarity fp*_a_* (2.1) and flow share fs*_a_* (2.2), we explore two contrasting cases: the *real-time scenario* or the *deferred scenario*, respectively, where the regulator has access to real-time data or deferred data, respectively, so fp*_a_* and fs*_a_* can be calculated based on 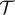 or based on 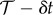 (with *δt* representing the delay of data collection), respectively. We set *δt* = 1 year, a deliberately large value for data collection. For each strategy explored, the relative decrease in the LSCC size is evaluated at increasing values of *F_n_*. For each value of *F_n_*, we keep track of the proxy for prevention effectiveness (the relative decrease in the LSCC size (2.4)), and of the four proxies for prevention costs: the two relative costs to the regulator (relative agent cost *F_n_* and flow cost 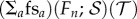); and two measures of market distortions (disruption to the overall flow polarity 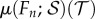 (2.5) and disruption to the overall flow share 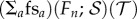).

## 3. Results and discussion

### 3.1. Analyses and representation of trade networks using market categories

**Figure 1.**
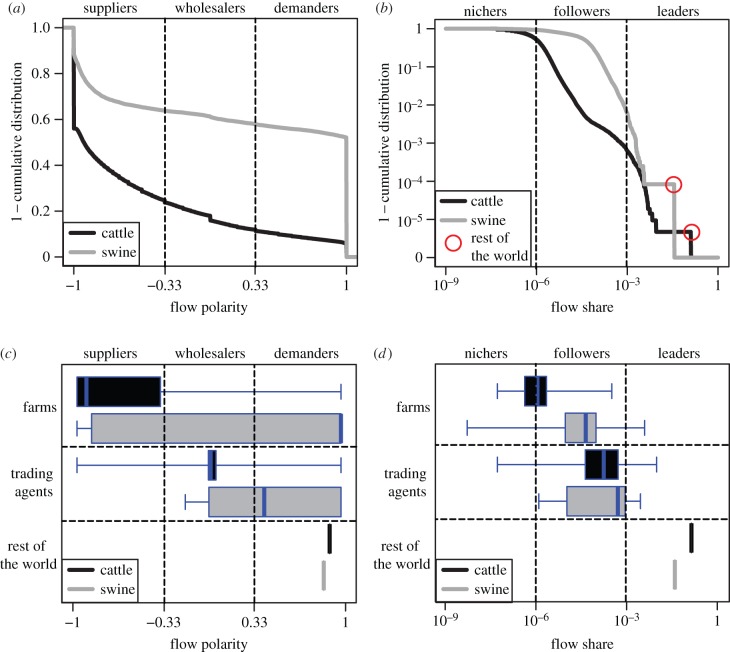
Categorization of economic agents: flow polarity and flow share. (*a*,*b*) Distributions of flow polarity and flow share in the cattle and swine markets. Flow polarity and flow share of an economic agent quantify its *position along the supply chain* and *market leadership*, respectively. Qualitatively, flow polarity (2.1) can be used to define *suppliers*, *wholesalers* and *demanders*, categories of agents corresponding to values that are negative, symmetrically distributed about zero, and positive, respectively. Similarly, flow share (2.2) can be used to define *nichers* (low values), *followers* (mid values) and *leaders* (high values). (*c*,*d*) Distributions of flow polarity and flow share per group of agents in the cattle and swine markets. Groups are either sets of French *farms* or *trading agents*, or a single entity aggregating all agents located outside of France, namely the *rest of the world*. Flow polarity and flow share are calculated over year 2009 for cattle and throughout year 2010 for swine.

**Figure 2.**
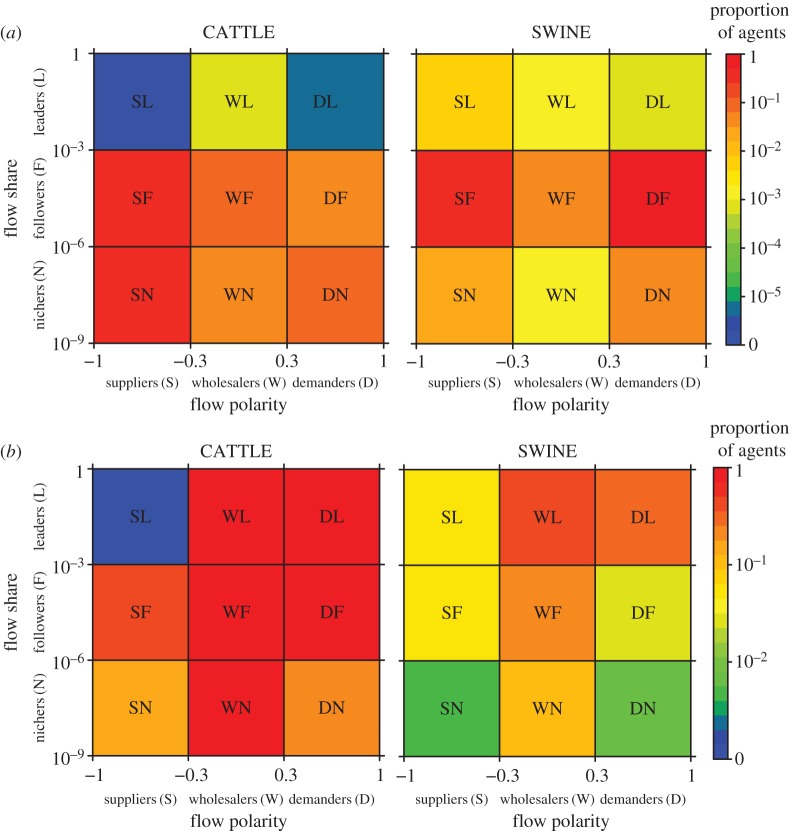
Market categories defined according to flow polarity and flow share and used to assess joint economic–epidemiological risks. (*a*) Proportion of agents in the whole population that are in each *market category*, i.e. agents with the given ranges of flow polarity and flow share. (*b*) Proportion of agents in the LSCC that are in each market category. The LSCC is a proxy for both the probability of an outbreak and the epidemic final size. The risk of outbreak increases as the number of agents that are in the LSCC increases. To ease interpretation, flow polarity and flow share are discretized on a 3 × 3 grid, leading to a total of nine market categories. The corresponding non-discretized marginal distributions of flow polarity and flow share for the cattle and swine markets are available in figure 1*a*,*b*. Flow polarity and flow share are calculated over year 2009 for cattle and year 2010 for swine.

Considering the French datasets for livestock movements of cattle and swine, we categorize agents with similar market characteristics, according to position along the supply chain, quantified by flow polarity (figure 1*a*,*c*), and market leadership, quantified by flow share (figure 1*b*,*d*). Agents with similar ranks in flow polarity and flow share belong to the same market category (figure 2*a*). Since market categories are relatively invariant over time (electronic supplementary material, section B.1, figure S1), we focus our subsequent analyses based on 2009 for cattle and 2010 for swine.

We notice suppliers and demanders are overly represented in the cattle and swine markets, respectively (figure 1*a*). Since demanders act as epidemiological dead-ends, our analyses suggest that the cattle market is riskier than the swine market, a result in agreement with [12]. According to the distribution of flow share, the swine market is less scattered than the cattle market (figure 1*b*). For instance, the median flow share is 10^−4.4^ in swine in 2010 and only 10^−6.0^ in cattle in 2009, i.e. the median flow share in the swine market is 40 times larger than in the cattle market. This indicates a larger economic risk associated with the failure of a typical swine agent compared with the failure of a typical cattle agent.

To shed light on the practical meaning of flow polarity and flow share, we analyse the correspondence between standard market groups and our market categories (figure 1*c*,*d*). For cattle, we remark that farms and trading agents correspond to suppliers and wholesalers, while for swine, farms and trading agents rather correspond to wholesalers-to-demanders (figure 1*c*). For both cattle and swine, French exchanges with the rest of the world essentially correspond to exports, i.e. the rest of the world acts as a strict demander. Concerning flow share for cattle and swine, farms and trading agents correspond to nichers-to-followers and followers-to-leaders, respectively (figure 1*d*). The rest of the world represents a considerable flow share (about 13% for cattle and 4% for swine) and can be described as a major market leader for both trade systems.

We compare the cattle and swine markets using the market categories defined in figure 2*a*. In the cattle market, the proportion of leaders is small, irrespective to the flow polarity. The most represented market categories are SN and SF, with no significant difference between nichers and followers. In the swine market, the largest proportions of agents are in the SF and DF market categories, illustrating the fact that followers exhibit a polarized activity most of the time. These distributions are modified when scrutinizing the LSCC. Indeed, for both cattle and swine, the proportion of agents belonging to the LSCC increases with increasing flow share (figure 2*b*), implying that the epidemiological risk associated with leaders is larger than that associated with nichers. This trend is confirmed by the distribution of average betweenness centrality among market categories (electronic supplementary material, section B.2, figure S2). For a given flow share, both risk indicators, i.e. the LSCC and betweeness centrality, typically have larger values for agents with negligible flow polarity, which suggests that wholesalers are probably stronger epidemiological drivers than suppliers, a finding in agreement with the theoretical results reported in [30,31]. We also notice, in line with [12], that the proportion of agents belonging to the LSCC is larger in cattle than in swine, which suggests that there is greater epidemiological risk associated with trade in cattle markets.

**Figure 3.**
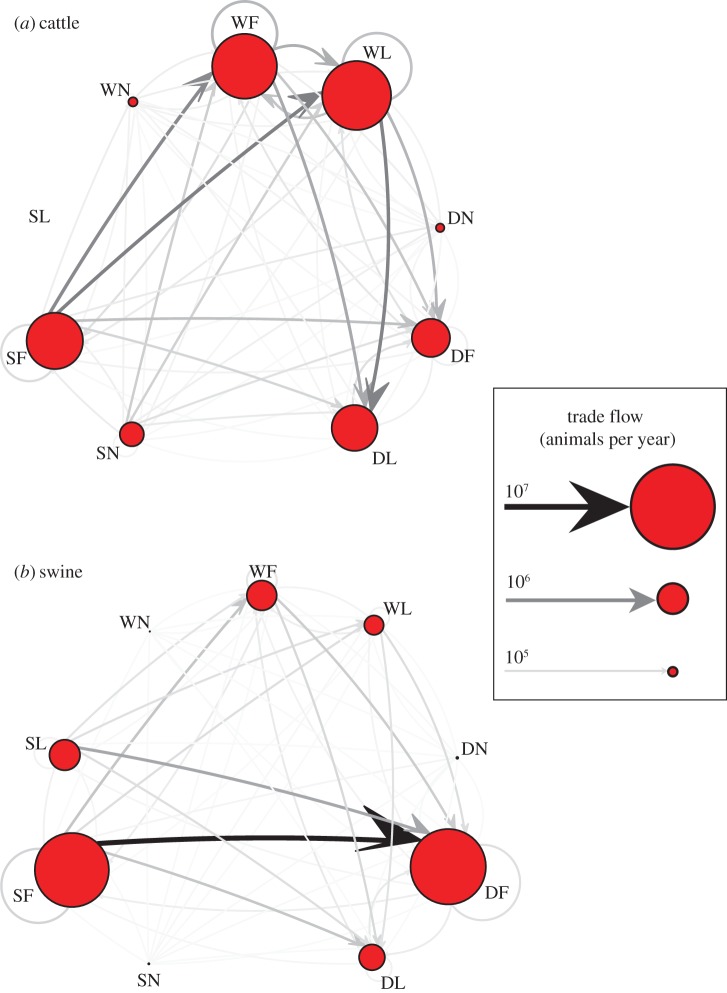
Trade networks of the cattle and swine livestock markets (*a*,*b*). Data are yearly aggregated (2009 for cattle and 2010 for swine). Each node encompasses all the agents in each market category, as defined on the 3 × 3 grid in figure 2. The size of each node is proportional to the yearly aggregated trade flow summed up over all agents in each market category. Widths and colour intensity of directed links (arrows) are proportional to corresponding yearly aggregated trade flow from/to each aggregated category, summed up over all the agents in the categories involved in in- or out-movements.

The use of market categories also enables comparison of connection patterns in the cattle and swine markets (figure 3). In the cattle market, the total-trade flow is relatively large for the WF and WL categories. Also, exchanges to and from wholesalers (irrespective of flow polarity) and within categories WF and WL are clearly over-represented in this market, thus generating structural loops and potentially infectious feedback. By contrast, there is a larger number of exchanges in the swine market than in the cattle market that involve direct transactions from suppliers to demanders, particularly from SF to DF, which leads to a trading structure with a limited number of potentially infectious feedback routes. Again, this result suggests that the swine market is less prone to epidemic spread compared with the cattle market.

### 3.2. Identification of targeted control strategies based on risk assessment per agent per market category

Particular preventive strategies are selected from preliminary risk assessments (§2.3.2). By definition, trade flow *per capita* increases with flow share for both cattle and swine, implying larger economic–epidemiological risks *per capita* associated with leaders compared to nichers. Flow polarity does not seem to influence trade flow significantly, especially in cattle (figure 3). Also note that in cattle, wholesalers have comparatively larger trade flows than the other categories. As in §3.1, this outcome suggests that wholesalers are stronger epidemiological drivers than suppliers.

At the scale of market categories, WL are more connected (figure 3), have a larger betweenness centrality (electronic supplementary material, section B.2) and are more likely to belong to the LSCC than SN (figure 2). This latter point renders WL representative both in the cattle and in the swine markets, although for the swine market WL agents are not involved in large volumes of trade (figure 3*b*). The swine market is driven mainly by trade flows from SF and SL to DF, which suggests that the epidemiological risk could be confined to only a few market categories. We therefore expect the cattle market to be at a greater epidemiological risk compared with the swine market, a finding in agreement with the results reported in §3.1 and in a previous study [28].

Taken together, our results corroborate, in agreement with the literature, that WL appear to act as infectious super-spreaders. WL, as market leaders, are also associated with major economic risks in case of failure. Like WL, SN can act as infection sources and are associated with epidemiological risk. However, in contrast with WL, SN have minor market importance and are less likely to induce market disruptions when disturbed. From a network perspective, WL (SN) can therefore be described as ‘hubs’ (anti-hubs), i.e. agents with a large (low) number of links compared with the average number of links per agent [37]. We therefore evaluate two contrasting strategies: the preferential targeting of hubs, i.e. WL, and the preferential targeting of anti-hubs, i.e. SN. The strategies targeting WL first (SN first) are referred to as *the WL strategies* (the *SN strategies*). In practice, we set *z*^suppliers^ = *z*^demanders^ = 1, *z*^nichers^ = 0 and *z*^leaders^ = 1 in (2.3) for the WL strategies, and *z*^suppliers^ = 1, *z*^demanders^ = 0, *z*^nichers^ = 1 and *z*^leaders^ = 0 in (2.3) for the SN strategies.

### 3.3. Evaluation of the targeted control strategies using multi-criteria decision analyses

Based on the results from the risk assessment (3.2), our MCDA focus on strategies preferentially targeting WL agents compared with strategies preferentially targeting SN agents. The relative performances of these strategies are compared for both markets using various criteria (as defined in §2.3.3).

**Figure 4.**
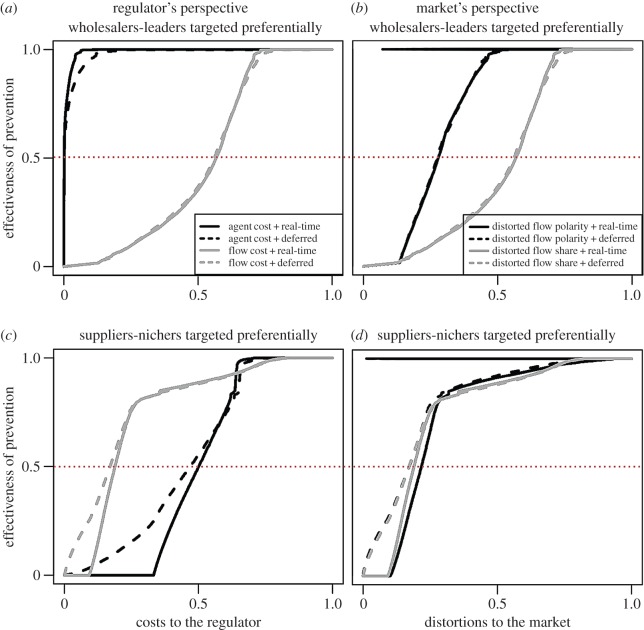
MCDA of contrasting targeted control strategies in the cattle livestock market. MCDA of strategies targeting WL first (*a*,*b*, with *z*^suppliers^ = 1; *z*^demanders^ = 1; *z*^nichers^ = 0; *z*^leaders^ = 1 in (2.3)) and SN first (*c*,*d*, with *z*^suppliers^ = 1; *z*^demanders^ = 0; *z*^nichers^ = 1; *z*^leaders^ = 0 in (2.3)). For each strategy, the *x*-axis quantifies prevention costs, i.e. the relative costs to the regulator (*a*–*c*) and relative distortions to the market (*b*–*d*) to reach a given prevention effectiveness against epidemics as depicted on the *y*-axis (e.g. the red dotted lines to reach 50% of prevention effectiveness). Prevention effectiveness is measured by the proportion of agents removed from the LSCC (2.4). Preventive strategies in the cattle market are implemented over year 2009. Market categories are defined either over 2009 (real-time information available on agents, plain curves) or over 2008 (deferred information available on agents, dashed curves). Each case corresponds to 100 replicate simulations (notice the weak variability).

**Figure 5.**
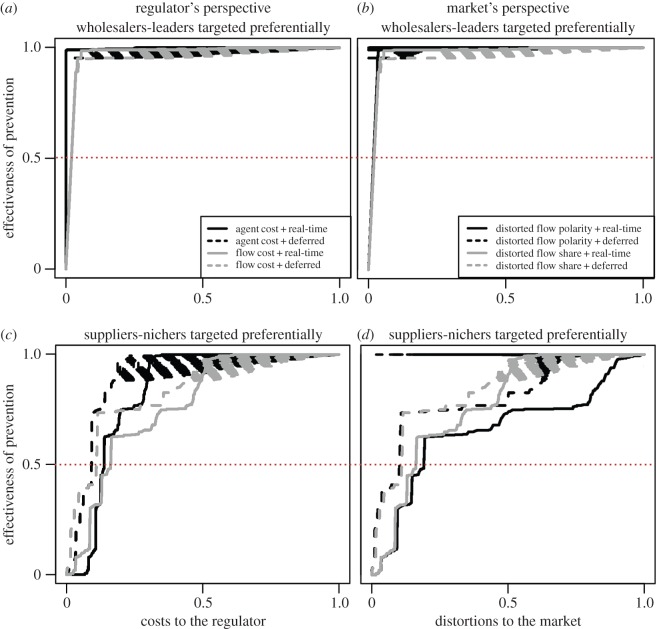
MCDA of contrasting targeted control strategies in the swine livestock market. Targeting strategies in the swine market are implemented over year 2010. Market categories are defined either over year 2010 (real-time information available on agents, plain curves) or over January 2010 (deferred information available on agents, dashed curves). The rest of the legend is identical to figure 4.

We start by introducing results from the regulator's point of view (i.e. quantifying agent costs and flow costs). WL strategies appear always to be more effective than SN strategies provided that the overall prevention cost is driven by the fraction of agents targeted (plain black curves in figures 4*a*,*c* and 5*a*,*c*). Under these conditions, we recover the commonly accepted wisdom that preferentially targeting the most central nodes in a heterogeneous network is the best way to mitigate an epidemic [17], where centrality of the nodes is determined by having large betweeness centrality and probability of belonging to the LSCC.

However, if the overall prevention cost is driven instead by the fraction of targeted flow, the SN strategies can be more efficacious than the WL strategies as seen in cattle (plain grey curves in figure 4*a*,*c*) but not in swine (plain grey curves in figure 5*a*,*c*). An example of a strategy driven by the fraction of targeted flow (total animals to be protected per time unit) would be an initiative whereby diagnostic tests would be distributed to farmers purchasing livestock in order to test biological samples from the animals purchased. This apparently counterintuitive result stems from the structure of the cattle network (figure 3*a*) and may not be so surprising: when applying tests at purchase, it may be better to cover both a large geographical and topological space (the ‘area’ covered by a network), i.e. to dispatch a constant number of tests to a very large number of premises with very small flow shares, rather than to a very small number of holdings with very large flow shares.

From the market's point of view, when targeting agents to prevent epidemics, the regulator will necessarily induce distortions to the market (figures 4*b*,*d* and 5*b*,*d*). Typical disruptions of the market include shifts in price, removal and later reintroduction of suppliers and demanders and/or local and global depletions in supply and demand stocks. Disruptions can be induced by infection and subsequent eradication of contaminated stock for sanitation or other preventive measures [28]. At first sight, it seems impossible to attain an optimal situation where epidemics are at low risk without affecting the market. Some strategies appear better than others though: while the SN strategies can induce fewer distortions than the WL strategies for most levels of prevention-effectiveness in cattle (figure 4*b*,*d*), the WL strategies are always the best in swine (figure 5*b*,*d*). These results suggest that cattle and swine markets, while both corresponding to heterogeneous livestock-exchange networks, require differing preventive measures.

Introduction of delays in the collection of data to design preventive strategies has little effect on our results (dashed versus plain curves in figures 4 and 5). Our conclusions are also not affected by the use of an alternative measurement of prevention effectiveness with an epidemiological risk proxy accounting for the time-varying nature of the network (i.e. the succession of transactions and hence of network links over time) nor by the inclusion of time lags in the implementation of preventive strategies (electronic supplementary material, section B.3).

Taken together, our results suggest that the WL strategies are not always the best. In particular for the cattle market, when costs of prevention are driven by the number of animals to target, the SN strategies perform better than the WL strategies.

## 4. Conclusion

Regulators tasked with managing disease outbreaks are generally constrained by limited resources [38]. Therefore, prioritizing interventions under limited resources is essential to achieve effective prevention and control of epidemic outbreaks. Typical targeted preventive measures include vaccination of risky agents, and risk-based surveillance such as blood-tests for identifying cryptic infectious cases, in particular for purchased animals in livestock-exchange markets. Targeted prevention is particularly relevant for a national regulator aiming to eradicate or control a disease in order to generate subsequent commercial benefits or to maintain a disease-free status to satisfy the requirements of the international animal health code. Acquiring or maintaining an internationally recognized disease-free status is associated with major benefits such as capacity to export livestock and reduction of control burdens. For instance, according to the FMA, surveillance measures of bovine tuberculosis, including on-farm visits and systematic animal testing, cost the French state as much as 20 million euros over the period 2010–2011 [39]. In this context, our analyses suggest that while the risk of epidemic introduction in France due to contaminated livestock imports appears limited, the economic risk associated with potential sanitary bans on French exports is important and could lead to major market disruptions. This outcome is consistent with a recent analysis of total-trade flows of livestock between EU countries [14].

While we build on an already rich literature that applies network analyses to inform health policies, in particular for the animal sector, we depart from existing studies by introducing a market-based categorization to analyse and protect trade networks propagating epidemics. Our market-based categorization, which we have found to be relatively stable over time, can intuitively describe market structure and interaction mechanisms. It can also be used to quantify joint economic–epidemiological risks, and hence to evaluate prevention strategies that target particular market categories, thereby concentrating resource application to confined sectors of the system at risk. In particular, when both the standpoints of the regulator and of the market are taken into account, we find that preferentially targeting SN, which are anti-hubs, can, in some cases, outperform the preferential targeting of WL, i.e. hubs. The preferential targeting of hubs appears to be systematically more effective when we only consider the regulator's point of view and assume that intervention costs are proportional to the number of economic agents to be protected. In summary, our study suggests that multiple perspectives should be adopted when evaluating targeted preventive strategies, a finding with general implications for epidemiological and ecological studies aiming at prioritizing interventions for maintaining healthy and diverse (eco-)systems.

Achieving the best epidemiological outcome under a constrained regulatory budget has been addressed by others, for example, through optimal control theory [40]. A typical objective of optimal control theory consists of finding an optimal amount of treatment at each time step to minimize the total number of infected animals during the course of an epidemic without exceeding a fixed budget. However, prior to the market-centric analyses introduced here, the influence of epidemics and subsequent regulatory measures on market functioning at the microeconomic scale were largely unknown [28]. Although we do not consider here a coupled dynamic model of infectious disease and economics dynamics (as proposed in a recent study based on theoretical modelling un-parametrized by data [23]), our study constitutes a first step towards understanding the likely impacts of epidemics on trade. At the interface between the data-motivated approach adopted here and the proof-of-concept approach exposed in [28], the elaboration of agent-based, economic–epidemiological models integrating temporal feedbacks will be the subject of future work. While we have focused our applications to animal health policy, our empirical formulation to identify market categories can aid the analysis of highly complex networks with multiple node types, and directed, weighted and dynamical links. We believe that the framework we have proposed can provide wider valuable insights to uncover the mechanisms underpinning joint disease and exchange dynamics.

## Supplementary Material

ESM to the manuscript

## Data accessibility

French livestock- and swine-exchange data were, respectively, provided by the French Ministry in charge of Agriculture (FMA) (contact point: bicma.sdspa.dgal@agriculture.gouv.fr) and the professional union BDPorc (contact point: administrateur@bdporc.fr), which operates under contract for the FMA. Data collection and analyses are subject to a confidentiality agreement available upon request from the designated contact points.
