# Supplementary material for: Market analyses of livestock trade networks to inform the prevention of joint economic and epidemiological risks
Source: J R Soc Interface. 2016 Mar;13(116):20151099. doi: 10.1098/rsif.2015.1099 (PMC4843675; doi:10.1098/rsif.2015.1099)
Supplement: ESM to the manuscript [file rsif20151099supp1.pdf]

Electronic Supplementary Material for the manuscript "Market analyses of livestock trade networks to inform the prevention of joint economic and epidemiological risk"

Mathieu Moslonka-Lefebvre[1,2,3,#], Christopher A. Gilligan[2], Hervé Monod[1], Catherine Belloc[4,5], Pauline Ezanno[4,5], João A. N. Filipe[2,6,*] and Elisabeta Vergu[1,*]

[1] MaIAGE, INRA, Université Paris-Saclay, 78350 Jouy-en-Josas, France
[2] Department of Plant Sciences, University of Cambridge, Downing Street, Cambridge CB2 3EA, United Kingdom
[3] AgroParisTech, F-75005 Paris, France
[4] INRA, UMR1300 Biologie, Epidémiologie et Analyse de Risques en santé animale, CS 40706, F-44307 Nantes, France
[5] LUNAM Université, Oniris, Ecole nationale vétérinaire, agroalimentaire et de l'alimentation Nantes-Atlantique, UMR BioEpAR, F-44307 Nantes, France
[6] Integrative Animal Science, School of Agriculture, Food and Rural Development, Newcastle University, Newcastle upon Tyne, NE1 7RU, UK
[#] Corresponding author, email: mathieu@moslonkalefebvre.com
[*] These authors contributed equally to this work

Contents

A Supplementary materials and methods **3**
A.1 Standard indicators of epidemiological risk: definitions and implications for epidemics . . . 3
A.2 Multiple-criteria decision analysis based on the average infection chain as a proxy for prevention-effectiveness . 3

B Supplementary results **4**
B.1 Temporal evolution of market categories . . . 4
B.2 Standard indicators of epidemiological risk in relation with market categories . . . 6
B.3 Multiple-criteria decision analysis of contrasted preventive strategies based on the average infection chain 7

A Supplementary materials and methods

A.1 Standard indicators of epidemiological risk: definitions and implications for epidemics

We define standard indicators to assess epidemiological risk for networks: the *proportion of agents belonging to the largest strongly connected component (LSCC)* and *betweenness centrality*.

The proportion of agents belonging to the LSCC is a standard proxy to assess both the probability of an outbreak and the epidemic final size [e.g. 1, 2]. Formally, the LSCC is the largest set of agents that can be reached by any other agent by following the direction of links over a time period $\mathcal{T}$. The larger the fraction of agents belonging to the LSCC, the larger the epidemiological risk. Since we calculate the proportion of agents belonging to the LSCC for each market category, we can estimate the contribution of each market category to the global epidemiological risk.

The betweenness centrality of an agent a, denoted BC_a, is the fraction of shortest path lengths that passes through a. The shortest path length from agent i to agent j is the smallest number of directed links needed to reach j from i. The larger BC_a, the larger the epidemiological risk of agent a [e.g. 3, 4]. Formally, BC_a, here normalised to account for networks differing in total number of agents, is given by:

$$\mathrm{BC}_a(\mathcal{T}) = \frac{1}{(N-1)(N-2)} \sum_{s \neq a \neq t} \frac{\sigma_{sat}(\mathcal{T})}{\sigma_{st}(\mathcal{T})} \,, \tag{1}$$

where $\sigma_{st}(\mathcal{T})$ and $\sigma_{sat}(\mathcal{T})$ are the number of shortest paths from agent s to agent t and the number of shortest paths from s to t passing through a during a time period $\mathcal{T}$ respectively. Since calculating shortest paths on large networks is computationally intensive, we approximate BC_a at order 3, i.e. we only consider shortest paths of length 3 or less.

A.2 Multiple-criteria decision analysis based on the average infection chain as a proxy for prevention-effectiveness

An alternative proxy to assess the effectiveness of preventive strategies: the average infection chain

We alternatively measure effectiveness, for differing strategies $\mathcal{S}$, as the *percentage decrease of the average infection chain* as function of the fraction of agents to target F_n. For a given time period $\mathcal{T} = [t_1; t_2]$, the out-going infection chain of a seller i (in-going infection chain of a demander j respectively) is the number of demanders that can be reached and hence infected from i by following temporally-compatible links (the number of sellers leading to j and from which j can hence be infected by following temporally-compatible links respectively). The out-going infection chain is calculated forwards in time (from t_1 to t_2), while the in-going infectious chain is calculated backwards in time (from t_2 to t_1). Both the out- and in- infection chains are epidemiological proxies [5, 6]. Following [7], we focus here on the average infectious chain, which is indifferently given, for a given $\mathcal{T}$, by the the average in-going infection chain or the average out-going infection chain as calculated over all nodes. In contrast with the largest strongly connected component, the average infection chain takes into account the sequentiality of the dates at which exchanges occur over period $\mathcal{T}$.

For each strategy explored, the percentage decrease of the average infection chain is evaluated at increasing values of F_n and we carry out 100 runs of random targeting.

Since we keep track of time, we further distinguish two types of interventions for a given $\mathcal{S}$: *early interventions* where all the F_n agents are surveilled at the beginning of the time period $\mathcal{T}$ where the strategy is evaluated, and *partly delayed interventions* where the first half of the ordered F_n agents is targeted at t_1 and the second half at $(t_1 + t_2)/2$.

Selection of geographical subsets of the cattle dataset to assess percentage decrease of the average infection chain

Even when carfully optimised, the evaluation of average infection chains remains computationally-intensive [8]. We hence restrict their calculations to the cattle market for small geographical scales, here French *Départements* (Dpt). We consider, on the one hand, exchanges occurring within the Ille-et-Vilaine Dpt and with the rest of the world (Dpt 35 + ROW) and, on the one hand, exchanges occurring within the Saône-et-Loire Dpt and the rest of the world (Dpt 71+ ROW). Dpt 35 and Dpt 71 are the largest French Dpt for dairy and beef production respectively (Tables S1-S2).

Table S1: **Overview of two contrasted *Départements* (Dpt) of the French cattle market in year 2009: Ille-et-Vilaine (Dpt 35; essentially dairy production) and Saône-et-Loire (Dpt 71; essentially beef production)**

quantity	Dpt 35 + ROW (dairy)	Dpt 71 + ROW (beef)
average number of animals exchanged per transaction	6.3	7.6
number of animals exchanged per year	600,889	597,918
number of transactions per year	95,051	78,385
number of agents (% in the LSCC)	6,853 (37%)	4,957 (63%)
number of strict suppliers	3,386	1,450
number of wholesalers	3,035	3,318
number of strict demanders	432	189

The LSCC is the largest strongly connected component.

Table S2: **Specialisation of farms in Dpt 35 and Dpt 71 in year 2009**

	% dairy farms	% mixed farms	% beef farms	% other farms	% trading agents
Dpt 35 (dairy)	**46.0**	28.0	9.6	16.0	0.4
Dpt 71 (beef)	3.7	17.5	**64.2**	13.5	1.1

B Supplementary results

B.1 Temporal evolution of market categories

We investigate to which extent market categories can be considered stable over time. For cattle, we explore temporal stability of market categories at a yearly scale for period 2005-2009 (Fig. S1). For swine, we only have access to deliveries occurring during year 2010, so we can not explore stability of market categories on a yearly scale.

Over years 2005-2009, flow polarity is more variable than flow share (Fig. S1A-B). Relative summaries such as flow share are expected to be highly stable over time and for any temporal scale used to calculate them [9]. Flow polarity (flow share) is roughly stable (extremely stable) from year 2008 to year 2009 (Fig. S1C-D). For both flow polarity and flow share, stability tends to increase for agents with negligible flow polarity and larger values of flow share, i.e. wholesalers-leaders (Fig. S1E-F).

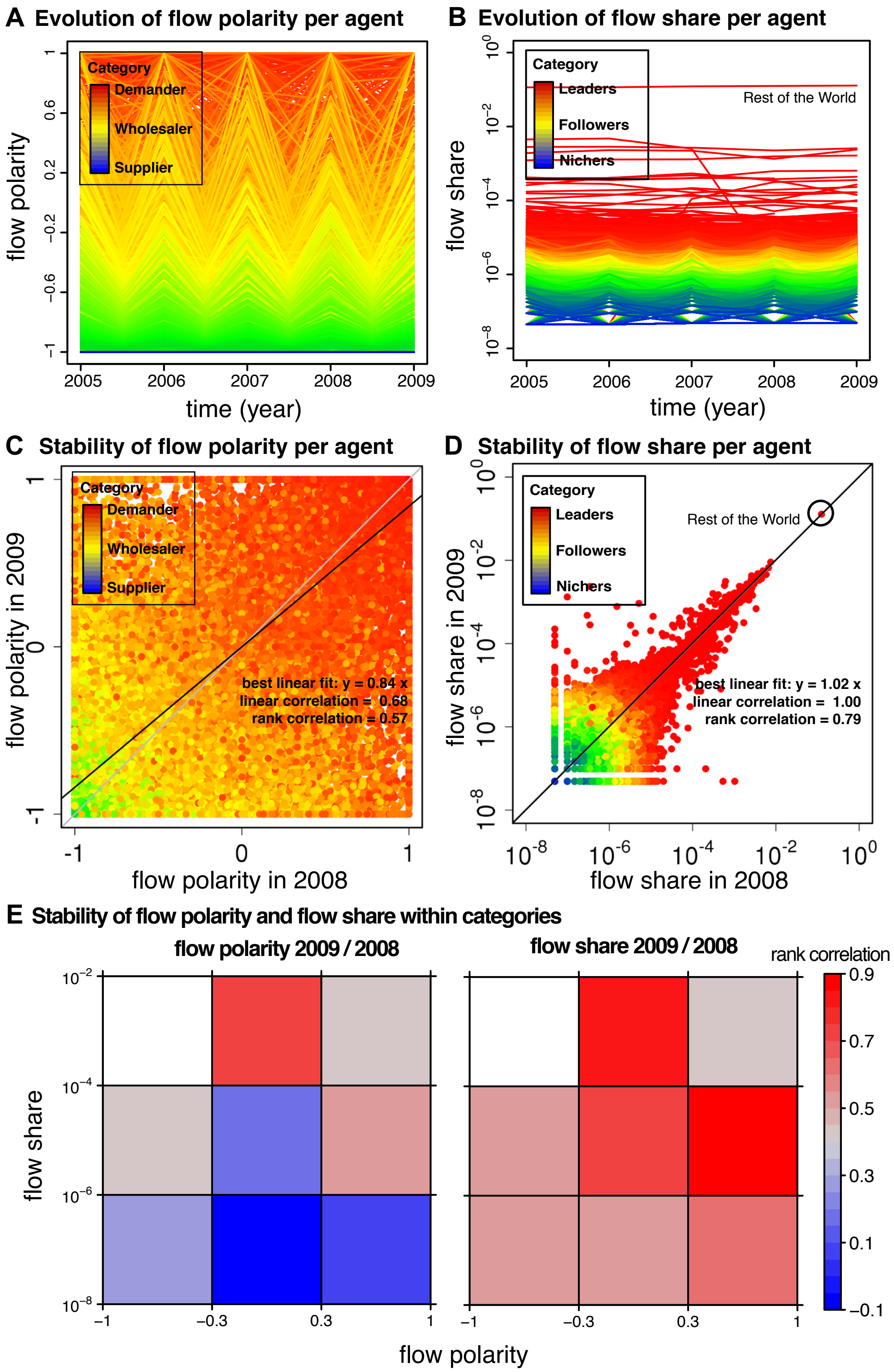

Figure S1: **Temporal stability of market categories for the cattle market. A)-B)** Annual evolution of flow polarity and flow share at the agent level from year 2005 to year 2009 for 5000 representative agents. Tenth of centiles in average annual values of flow polarity and flow share are used to cluster agents by color classes, from blue (minimal values) to red (maximal values). 5 agents are selected uniformly at random for each tenth of centile leading to a total of 5000 individual dynamics (about 2 % of total trajectories). **C)-D)** Stability of flow polarity and flow share at the agent level between years 2008 and 2009 for all agents. The color code is identical to (A-B). The black (grey) line represent the best linear fit $y = ax$ (the line $y = x$). Stability is assessed with Pearson's linear correlation coefficient and Spearman's rank correlation coefficient. **E)** Stability of flow polarity (left panel) and flow share (right panel) within market categories between years 2008 and 2009 for all agents. White cells correspond to empty categories. Market categories are defined here based on annual averages over five years and excluding exchanges with the rest of the world. Stability is assessed both with Pearson's linear and Spearman's rank correlation coefficients.

B.2 Standard indicators of epidemiological risk in relation with market categories

In addition to the proportion of agents belonging to the largest strongly connected component (Fig. 2 in the main text), we calculate the *betweenness centrality*, an additional standard indicator for epidemiological risk for both cattle and swine markets. We show how this measure scales with market categories, i.e. with flow polarity and flow share. For both cattle and swine, the betweenness centrality increases with flow share (Fig. S2), implying a larger epidemiological risk associated with leaders compared with nichers. For a given flow share, the average betweenness centrality tends to be larger for agents with negligible flow polarity: wholesalers are probably stronger epidemiological drivers than suppliers, a finding in agreement with the theoretical results reported in [10, 11].

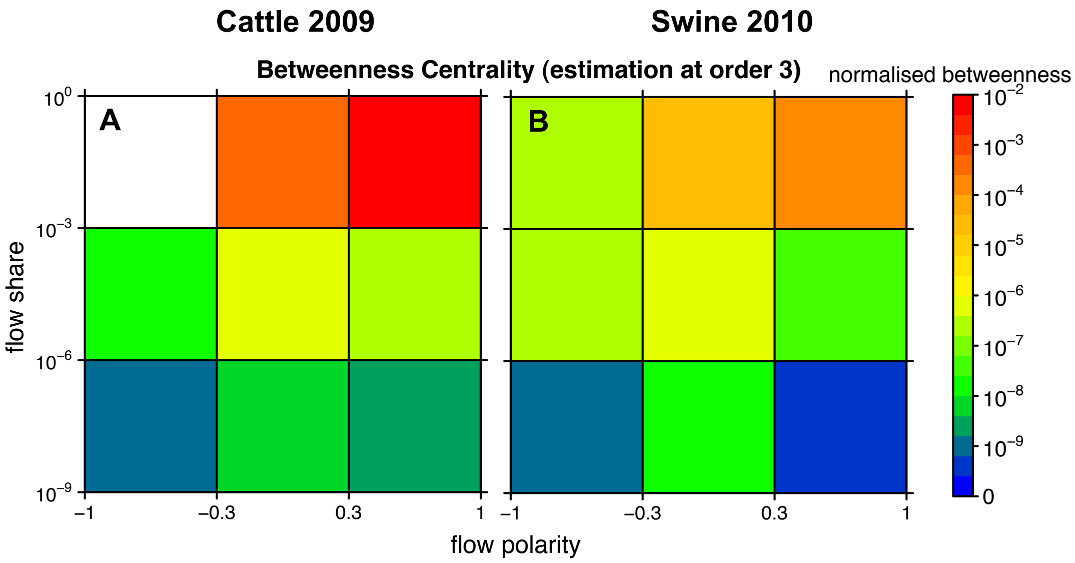

Figure S2: **Betweenness centrality as an indicator of epidemiological risk in the cattle and swine markets. A)-B)** Average normalised betweenness centrality per agent per market category. Betweenness centrality, here approximated at order 3, is considered a good indicator of epidemiological risk. The larger the betweenness centrality, the larger the risk. Market categories are defined as in Fig 2 of the main text. The white cell corresponds to an empty category.

B.3 Multiple-criteria decision analysis of contrasted preventive strategies based on the average infection chain

Measuring prevention-effectiveness based on the largest strongly connected component, we find, by carrying-out percolation experiments on the cattle market (Fig. 4 in the main text), that targeting suppliers-nichers first (SN strategy) induces lower relative flow-cost to the regulator and lower market distortions than targeting wholesalers-leaders first (WL strategy).

Here we test whether these findings stand when prevention-effectiveness is rather measured based on the average infection chain (Section A.2). We also explore impacts of delaying interventions. We focus our analyses on two contrasted geographical subsets of the French cattle market called Départements (Dpt): Dpt 35 (essentially dairy production) and Dpt 71 (essentially beef production). We also consider, for each Dpt, exchanges with the Rest Of the World (ROW).

The results remain qualitatively unchanged compared to those reported in the main text for the cattle market (Fig. 4 in the main text): the SN strategy outperforms the WL strategy as far as the relative flow-cost to the regulator and market distortions are concerned (Figs S3,S4,S5,S6). As expected, the WL strategy performs better than the SN strategy regarding relative agent-cost. Delaying interventions has a negligible effect on WL strategies and only affect SN strategies for very large values of prevention-effectiveness (Fig S3 versus Fig S4 and Fig S5 versus Fig S6)

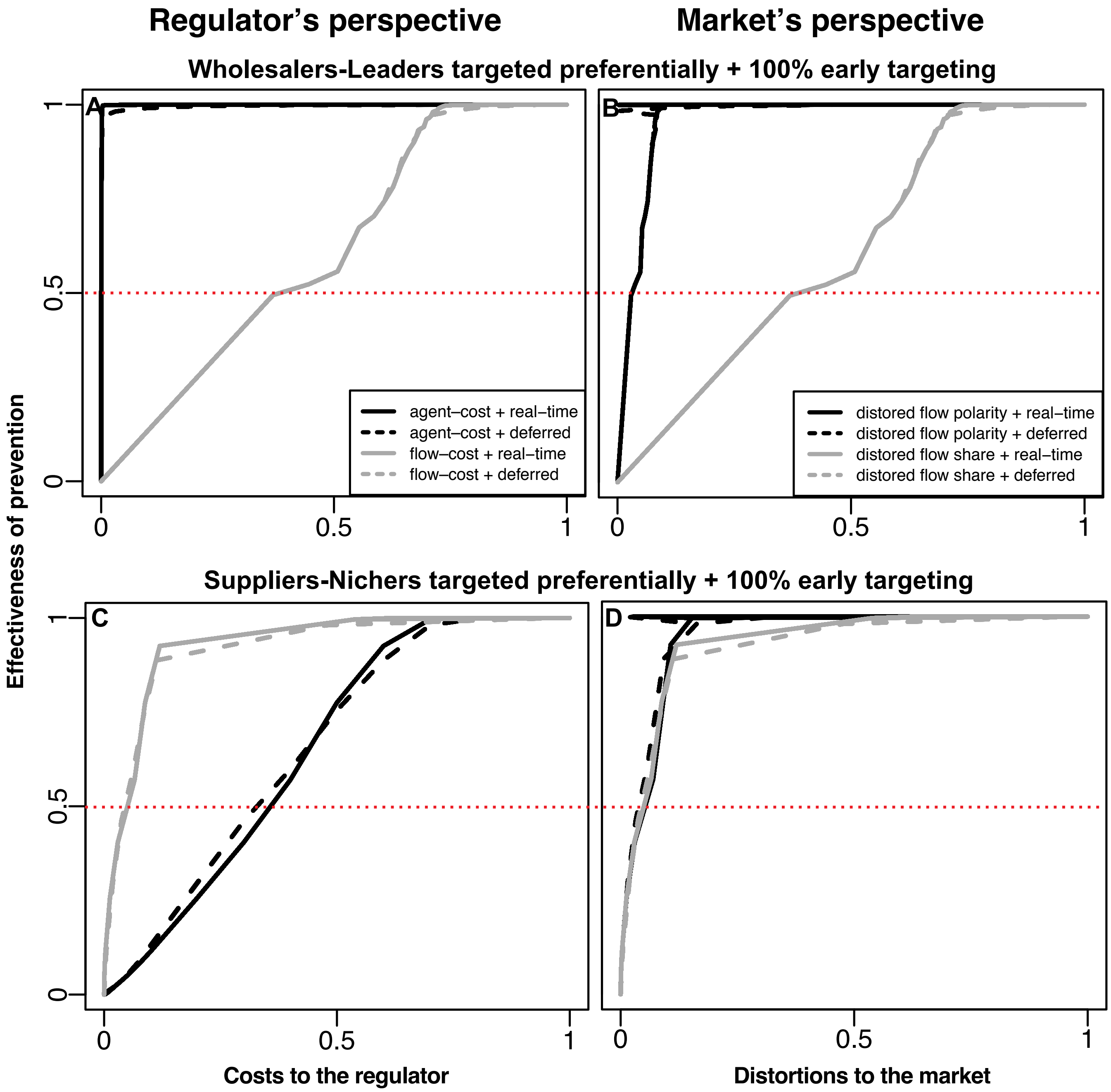

Figure S3: **Multiple-criteria decision analyses (MCDA) of contrasting targeted control strategies in Dpt 35 + ROW with early interventions.** MCDA of strategies targeting wholesalers-leaders first (**A**-**B**, with $z^{suppliers} = 1$; $z^{demanders} = 1$; $z^{nichers} = 0$; $z^{leaders} = 1$ in (3) of the main text) and suppliers-nichers first (**C**-**D**, with $z^{suppliers} = 1$; $z^{demanders} = 0$; $z^{nichers} = 1$; $z^{leaders} = 0$ in (3) of the main text). For each strategy, the x-axis quantifies prevention-efforts, i.e. the relative costs to the regulator (**A**-**C**) and relative distortions to the market (**B**-**D**) to reach a given prevention-effectiveness against epidemics as depicted on the y-axis (e.g. the red dotted lines to reach 50% of prevention-effectiveness). Prevention-effectiveness is measured by the relative decrease in the mean infection chain (Section A.2). We focus on Dpt 35 and associated exchanges with the rest of the world (ROW). Preventive strategies are implemented early, i.e at the beginning of year 2009, and evaluated over year 2009. Market categories are defined either over 2009 (real-time information available on agents, plain curves) or over 2008 (deferred information available on agents, dashed curves). Each case corresponds to 100 replicate simulations (notice the weak variability).

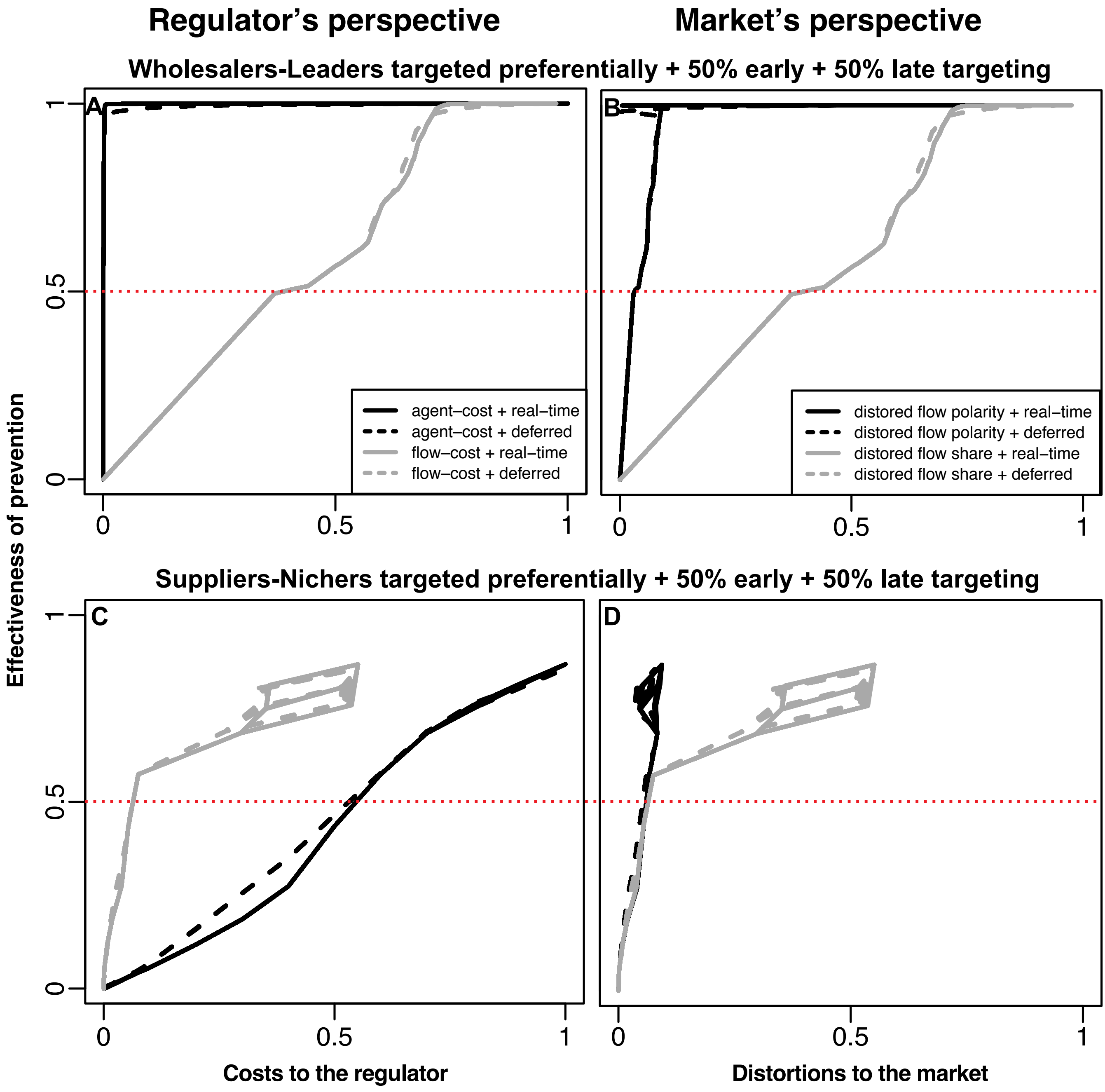

Figure S4: **MCDA of contrasting targeted control strategies in Dpt 35 + ROW with partly delayed interventions** We focus on Dpt 35 + ROW. Preventive strategies are implemented with a partial delay, i.e. the first half of agents to target are protected at the beginning of year 2009 and the second half on mid-2009, and evaluated over year 2009. The rest of the legend is identical to Fig. S3.

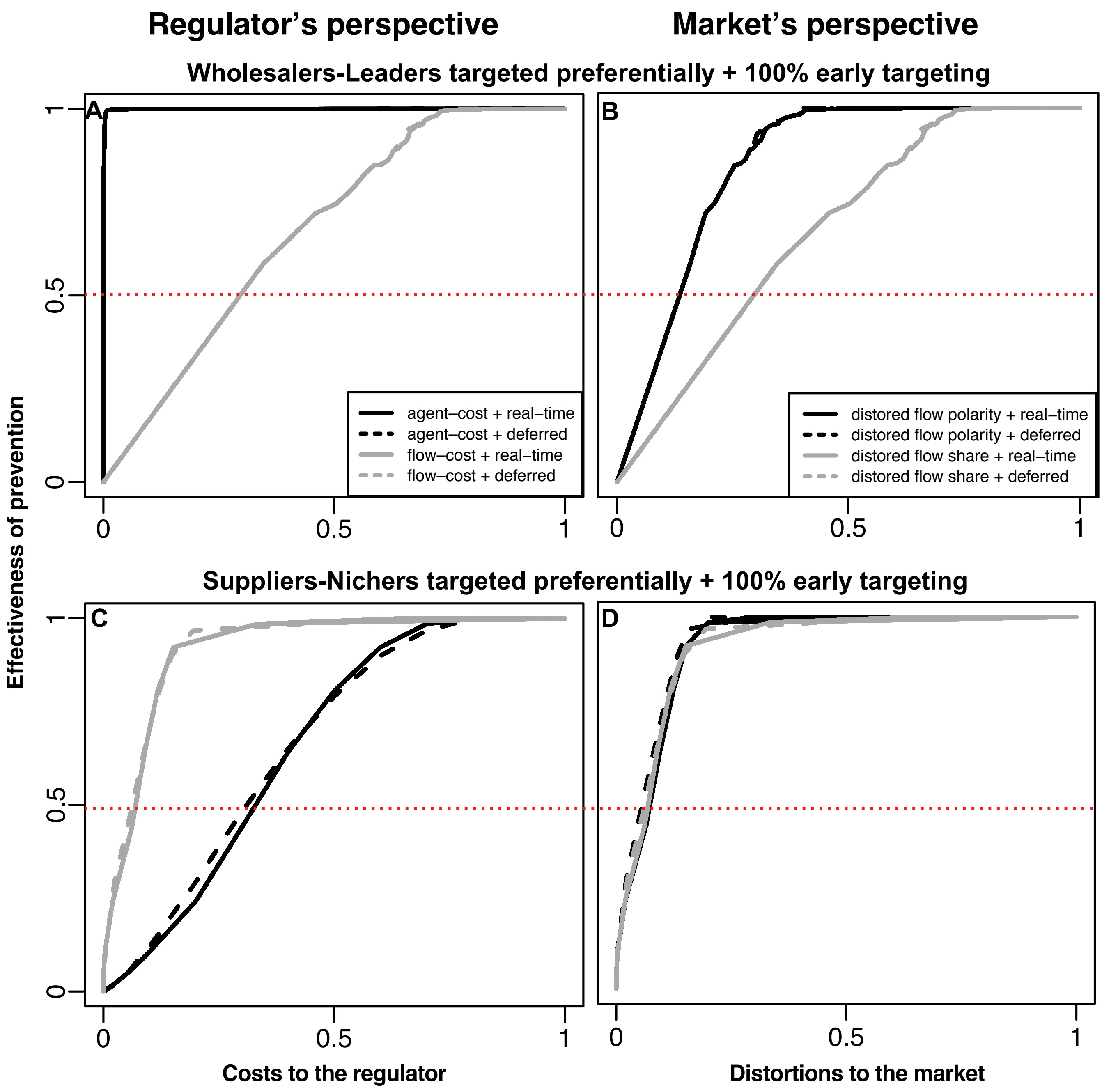

Figure S5: **MCDA of contrasting targeted control strategies in Dpt 71 + ROW with early interventions** We focus on Dpt 71 + ROW. Preventive strategies are implemented early, i.e. at the beginning of year 2009, and evaluated over year 2009. The rest of the legend is identical to Fig. S3.

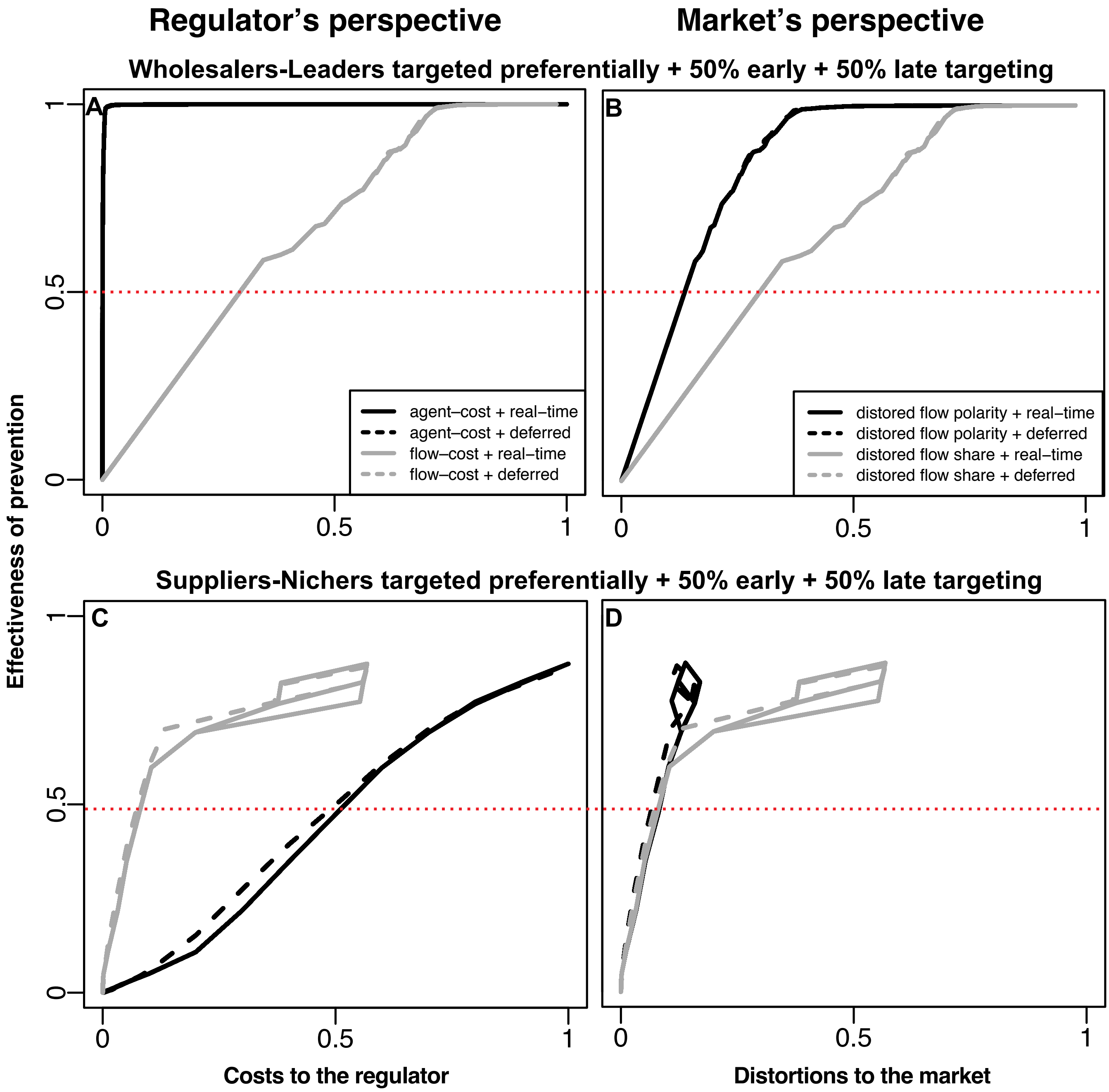

Figure S6: **MCDA of contrasting targeted control strategies in Dpt 71 + ROW with partly delayed interventions** We focus on Dpt 71 + ROW. Preventive strategies are implemented with a partial delay, i.e. the first half of agents to target are protected at the beginning of year 2009 and the second half on mid-2009, and evaluated over year 2009. The rest of the legend is identical to Fig. S3.

References

[1] Meyers LA, Newman MEJ, Pourbohloul B. Predicting epidemics on directed contact networks. Journal of Theoretical Biology. 2006;240(3):400–418.

[2] Rautureau S, Dufour B, Durand B. Structural vulnerability of the French swine industry trade network to the spread of infectious diseases. Animal. 2012;6(07):1152–1162.

[3] Brandes U. A faster algorithm for betweenness centrality. The Journal of Mathematical Sociology. 2001;25(2):163–177.

[4] Buttner K, Krieter J, Traulsen A, Traulsen I. Static network analysis of a pork supply chain in Northern Germany: characterisation of the potential spread of infectious diseases via animal movements. Preventive Veterinary Medicine. 2013;110:418–428.

[5] Dube C, Ribble C, Kelton D, McNab B. Comparing network analysis measures to determine potential epidemic size of highly contagious exotic diseases in fragmented monthly networks of dairy cattle movements in Ontario, Canada. Transboundary and Emerging Diseases. 2008;55(9-10):382–392.

[6] Noremark M, Hakansson N, Lewerin SS, Lindberg A, Jonsson A. Network analysis of cattle and pig movements in Sweden: measures relevant for disease control and risk based surveillance. Preventive Veterinary Medicine. 2011;99:78–90.

[7] Lal Dutta B, Ezanno P, Vergu E. Characteristics of the spatio-temporal networks of cattle movements in France over a 5-year period. Preventive Veterinary Medicine. 2014;In press (doi: 10.1016/j.prevetmed.2014.09.005).

[8] Noremark M, Widgren S. EpiContactTrace: an R-package for contact tracing during livestock disease outbreaks and for risk-based surveillance. BMC Veterinary Research. 2014;10(1):71.

[9] Perra N, Goncalves B, Pastor-Satorras R, Vespignani A. Activity driven modeling of time varying networks. Sci Rep. 2012;2.

[10] Pautasso M, Xu X, Jeger MJ, Harwood TD, Moslonka-Lefebvre M, Pellis L. Disease spread in small-size directed trade networks: the role of hierarchical categories. Journal of Applied Ecology. 2010;47(6):1300–1309.

[11] Moslonka-Lefebvre M, Harwood T, Jeger MJ, Pautasso M. SIS along a continuum (SIS_c) epidemiological modelling and control of diseases on directed trade networks. Mathematical Biosciences. 2012;236(1):44–52.

Running title: livestock trade networks and economic-epidemiological risk
